# Capsaicin- resistant arterial baroreceptors

**DOI:** 10.1186/1477-5751-5-6

**Published:** 2006-05-18

**Authors:** Patrick J Reynolds, Wei Fan, Michael C Andresen

**Affiliations:** 1 Department of Physiology and Pharmacology, Oregon Health & Science University, Portland, Oregon 97239-3098, USA; 2 Vollum Institute, Oregon Health & Science University, Portland, Oregon 97239-3098, USA

## Abstract

**Background:**

Aortic baroreceptors (BRs) comprise a class of cranial afferents arising from major arteries closest to the heart whose axons form the aortic depressor nerve. BRs are mechanoreceptors that are largely devoted to cardiovascular autonomic reflexes. Such cranial afferents have either lightly myelinated (A-type) or non-myelinated (C-type) axons and share remarkable cellular similarities to spinal primary afferent neurons. Our goal was to test whether vanilloid receptor (TRPV1) agonists, capsaicin (CAP) and resiniferatoxin (RTX), altered the pressure-discharge properties of peripheral aortic BRs.

**Results:**

Periaxonal application of 1 μM CAP decreased the amplitude of the C-wave in the compound action potential conducting at <1 m/sec along the aortic depressor nerve. 10 μM CAP eliminated the C-wave while leaving intact the A-wave conducting in the A-δ range (<12 m/sec). These whole nerve results suggest that TRPV1 receptors are expressed along the axons of C- but not A-conducting BR axons. In an aortic arch – aortic nerve preparation, intralumenal perfusion with 1 μM CAP had no effect on the pressure-discharge relations of regularly discharging, single fiber BRs (A-type) – including the pressure threshold, sensitivity, frequency at threshold, or maximum discharge frequency (n = 8, p > 0.50) but completely inhibited discharge of an irregularly discharging BR (C-type). CAP at high concentrations (10–100 μM) depressed BR sensitivity in regularly discharging BRs, an effect attributed to non-specific actions. RTX (≤ 10 μM) did not affect the discharge properties of regularly discharging BRs (n = 7, p > 0.18). A CAP-sensitive BR had significantly lower discharge regularity expressed as the coefficient of variation than the CAP-resistant fibers (p < 0.002).

**Conclusion:**

We conclude that functional TRPV1 channels are present in C-type but not A-type (A-δ) myelinated aortic arch BRs. CAP has nonspecific inhibitory actions that are unlikely to be related to TRV1 binding since such effects were absent with the highly specific TRPV1 agonist RTX. Thus, CAP must be used with caution at very high concentrations.

## Background

Sensory information from visceral organs enters the central nervous system via separate pools of primary afferents that synapse at either the spinal cord or the brain stem [1,2]. Cranial primary afferents that synapse first within the brain stem have cell bodies in the nodose ganglia (NG) and share many general morphological, cellular and molecular properties with the somatic and visceral sensory neurons that send information to the spinal cord and whose cell bodies lie in the spinal dorsal root ganglia (DRG) [3]. On the basis of the structure of their peripheral axons and conduction velocities, spinal and cranial sensory neurons are divided into two broad subdivisions as myelinated (A-fiber) or unmyelinated (C-fiber) phenotypes [4]. A key membrane channel protein associated with C-type sensory neurons is the vanilloid receptor TRPV1 (or VR1) [5], although lightly myelinated, A-δ spinal sensory neurons may also express TRPV1 [6-8]. Surveys of mRNA suggest that TRPV1 is expressed in over 80% of NG as well as DRG neurons [9]. The functional significance of TRPV1 in NG neurons is generally unclear, although in gastrointestinal vagal afferents is associated with gastric mucosal acid exposure [10].

Arterial baroreceptors (BRs) are a specific group of cranial afferent neurons that have structurally primitive mechanically sensitive endings within the adventitial layer of large central arteries such as the aortic arch [11,12]. Aortic BRs with A- and C-type axons have distinctive pressure-discharge properties [13]. The rat aortic depressor nerve can be isolated *in vitro *in an aortic arch preparation and highly stable recordings made from single fiber aortic arch BRs[14]. Here, our goal was to test under highly controlled conditions in vitro whether the compound action potentials and pressure-discharge relations of aortic BRs were affected by TRPV1 activation. Using two selective ligands, CAP and resiniferatoxin (RTX), the data suggest that myelinated, regularly discharging A-type BRs are highly CAP-resistant but that CAP must be used with caution at very high concentrations even with A-type BRs.

## Results

### CAP blocks C-fiber volley of aortic depressor nerve trunk

**Figure 1:**
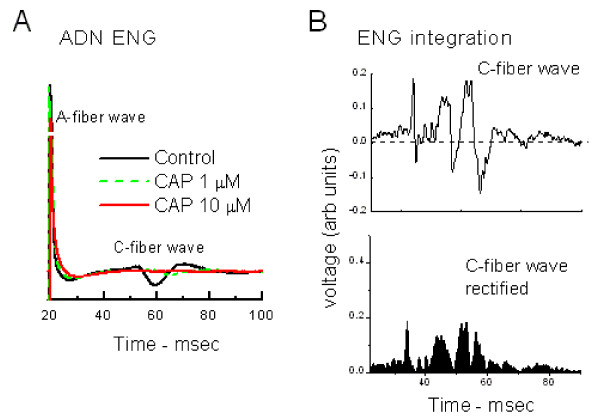
Capsaicin depressed the C-wave recorded from the whole nerve trunk of the aortic depressor nerve (ADN). The ADN electroneurogram (ENG) had an early arriving A-wave and a late broad, C-wave (panel A). This representative ADN was estimated to be 21 mm in length. Application of a pledget soaked in saline (Control), 1 μM CAP (green, broken) and then 10 μM CAP (red) for ten min at each condition resulted in progressive depression and then elimination of the slow conducting (0.5 m/sec) C-wave while the fast conducting, A-wave was preserved. Note that the y-axis is broken to better display the C-wave. Amplitude units are arbitrary since the degree of electrical isolation varied from nerve to nerve and was affected by saline shunting and the length of free nerve ending. For semi-quantitative comparisons across nerves, segments of the ENG were full wave rectified and digitally integrated to represent the amplitude of the entire wave (panel B, lower).

The nerve trunk of the aortic depressor nerve (ADN) runs separately as a distinct axon bundle from the sensory arbors of the individual BRs at the aortic arch until merging with the vagal trunk near the NG. Cutting both ends of the ADN (peripheral and aortic arch ends) and stripping them of their outer sheath provided sufficient length of nerve to stimulate and record the conducted whole nerve compound action potential [15]. At the lowest stimulation intensities, shocks to the ADN elicited an early arriving, very short duration compound action potential. Increasing the intensity of the electrical stimulus shock added a broad, slowly conducted, compound spike following the sharp, early spike (Figure 1). Supramaximal stimulation intensities activated these two, distinct volleys with the early arriving spike corresponding to calculated conduction velocities of between 8 and 12 m/sec (n = 6) that were within the Aδ-fiber range of conduction (Figure 1A). The late arriving and often more complex volley (Figure 1B) had lower amplitude and much longer duration – often 20–40 msec – and the leading edge of the late wave corresponded to a calculated conduction velocity of not greater than 1 m/sec in all cases (n = 6). Such evoked responses were stable in control experiments over periods of >30 min. Addition of 1 μM CAP (Figure 1A) at a point along the ADN trunk between the stimulating and the recording electrodes substantially reduced the C-fiber volley amplitude and delayed its arrival in time after 15 min exposure. To facilitate comparison, ENG signals were full wave rectified and then integrated to express complex waveform magnitudes (Figure 1B). On average (n = 4), 1 μM CAP did not significantly alter the A-fiber volley integral (0.68 ± 0.17 vs. 0.88 ± 0.18; p > 0.05), but significantly depressed the C-fiber volley (1.38 ± 0.16 vs. 0.51 ± 0.16, p < 0.02). Thus, aortic C-fiber BR axons in the ADN trunk are CAP-sensitive and the A-fiber axons are CAP-resistant.

### Single fiber baroreceptor responses to CAP

**Figure 2:**
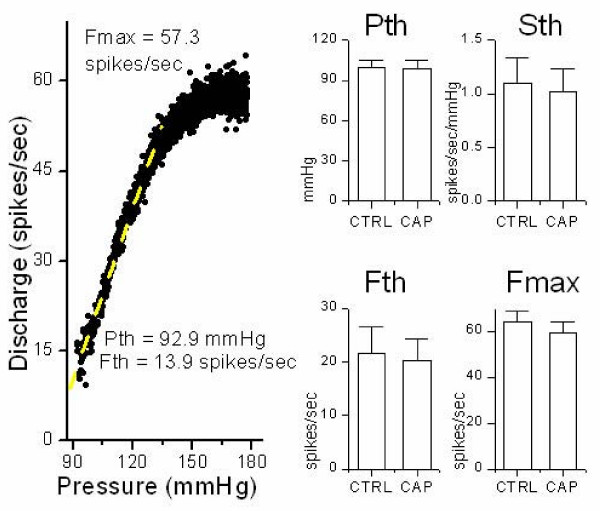
*Capsaicin-resistant baroreceptors*. Capsaicin effects on the pressure-discharge relations of regularly discharging, single-fiber, aortic BRs were assessed by comparing four key discharge parameters. Pressure threshold (Pth) and threshold frequency (Fth) were calculated as the average pressure and frequency (respectively) for the first 10 discharge points. The slope sensitivity (Sth) was based on a least squares, linear regression fit of the discharge points over the initial 20–40 mmHg of the relation (white dashed line). The maximum discharge (Fmax) averaged the discharge frequency sampled from the highest 20 mmHg for that unit – generally >200 points. The classification as regularly discharging was based on a moderate (<%) coefficient of variation (COV) of discharge, high Fmax (>50 Hz) and high slope-sensitivity (>0.5 spikes/sec/mmHg) – all characteristic of A-fiber BRs. 1 μM CAP for 15 min failed to alter the pressure-discharge response of regularly discharging BRs (n = 10) or their mean discharge parameters. The COV was calculated using this same sample and for this BR was 2.94%. Linear regression fit (Y = A + B * X) yielded A=-75.0 ± 0.9, B = 0.95 ± 0.01, R = 0.99 ± 1.68, n = 470 points, p-value<0.0001. Averages (± SEM) of these derived parameters for Pth, Sth, Fth and Fmax.

In order to test pressure activation of single fiber aortic BRs under highly controlled conditions, we isolated the ADN together with the aortic arch in an *in vitro *preparation[14,16,17]. Split fibers containing single active BR units were dissected from the nerve trunk and subjected to slow ramp increases in aortic arch pressure while recording the nerve discharge. The most common pressure-discharge relation for single fiber BRs showed a very tight band of highly regular discharge (Figure 2). Such BRs had relatively low absolute threshold values (Pth), a sharp jump in frequency at threshold from zero to 15–20 spikes/sec (Fth) and maximum discharge rates of 50–60 Hz (Fmax). The calculated coefficient of variation (COV) for regularly discharging BRs was 3.2 +/- 1.4%, n = 9. These discharge characteristics are typical of myelinated aortic BRs [13,18]. Mean perfusion pressure was held at 80 mmHg to control for acute resetting [19] and under these conditions the pressure-discharge relations were superimposable over the full control periods of at least 15 min. Perfusion of the lumen of the aortic arch with 1 μM CAP did not alter any aspect of the pressure-discharge properties of such regularly discharging BRs even after 15 min (Figure 2). On average (n = 10), the discharge properties of regularly discharging BRs were unaltered by 1 μM CAP (Figure 2). CAP did not alter Pth (p = 0.79), Sth (p = 0.80), Fth (p = 0.84) or Fmax (p = 0.57). Thus, at concentrations of CAP that interrupted C-wave axonal transmission in the ADN trunk, the responses of regularly discharging BRs to pressure were unaffected. This 1 μM concentration of CAP is close to the K_D _(2 μM) reported for DRG neurons[20].

**Figure 3:**
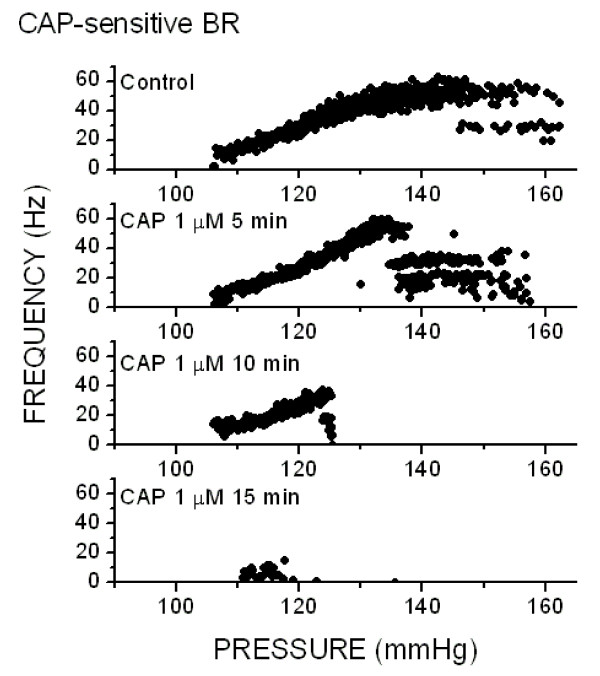
*Capsaicin-sensitive baroreceptor*. Capsaicin (1 μM) depressed the pressure-discharge response of an irregular aortic BR. The discharge properties including high coefficient of variation (COV = 9.9) of discharge, low discharge rate (maximum of 40 Hz) and low slope-sensitivity – all characteristic of C-fiber BRs. Pressure was ramped from 40 – 160 mmHg at a rate of 1 mmHg/sec. CAP progressively interrupted discharge with intermittent drops in discharge rates to half or less the prevailing rate (points at high pressures falling below the general trend). At 10 min and beyond, the BR failed to respond to suprathreshold pressures and at 15 min the relation was completely blocked. The loss of individual instantaneous frequency points by discrete increments (integer reciprocals, e.g ½. 1/3, etc.) indicates likely conduction block.

Routine testing of active single BR fibers used ramped pressure tests to 160–180 mmHg in an attempt to record C-fiber BRs. C-fiber BRs generally require relatively higher pressures for activation than for regularly discharging A-type BRs [13,18]. One single fiber BR had a relatively high Pth (106 mmHg) and irregular discharge under control conditions (Figure 3). Discharge began at near zero and only gradually increased as pressure ramped higher. The irregular discharge produced a scattered band of points as pressure increased. At the upper levels of firing, the broad irregularity produced a COV of 9.9% – over three fold greater than the regular discharge group and a value highly unlikely to belong to the regular discharging BR group (p < 0.02). The low and irregular discharge rate plus the low value for the slope sensitivity (Sth) are characteristic of rat C-type BRs [13,18]. Addition of 1 μM CAP to the perfusate (Figure 3) rapidly depressed the pressure discharge relation beginning at 5 min exposure (Figure 3). The BR failed to sustain discharge at high pressures but discharge near threshold was generally similar to control. By 10 min in CAP, discharge from this BR was no longer sustained above 125 mmHg. At 15 min in 1 μM CAP, the BR was nearly inexcitable and only fired a scattering of action potentials to aortic arch pressures above its control threshold level (Figure 3). This inhibition of discharge was partially reversed following 30 min of CAP-free wash. Thus, CAP eliminated the response to pressure of this irregularly discharging BR.

### Capsaicin but not RTX non-specific depresses BR discharge at high concentrations

**Figure 4:**
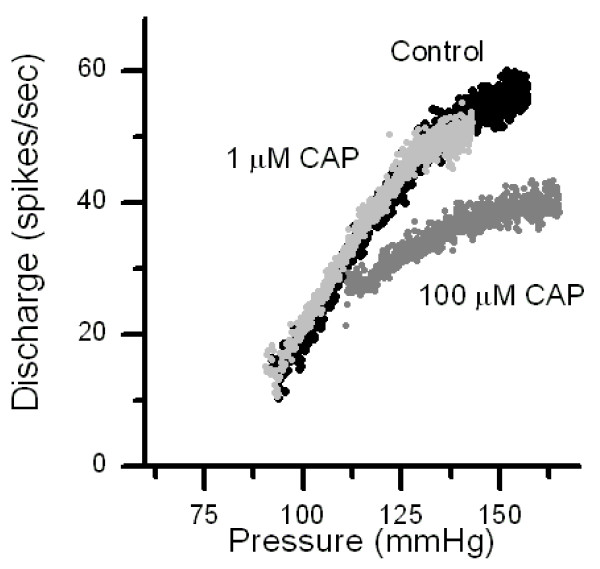
Non-specific actions of CAP on a capsaicin-resistant BR. 1 μM CAP for 15 min (light gray points) failed to alter the pressure-discharge response of this regularly discharging BR (control, black points). Raising CAP to 100 μM depressed the slope of the discharge relation at 15 min exposure (dark gray points).

Reports suggest that CAP has additional, non-specific effects at concentrations that are well beyond saturation of specific binding sites at TRPV1 [20]. Given the high binding specificity of these agonists for TRPV1 [20], we considered concentrations 100 fold greater than the known receptor Kd as potentially nonspecific. We tested much higher concentrations of CAP in otherwise CAP-resistant, A-type BRs (# 10 μM, n = 6). At concentrations >10 μM, CAP modified the pressure-discharge relations in a distinctly different manner than observed in the irregularly discharging CAP-sensitive BR (Figure 3). 100 μM CAP substantially increased the Pth and depressed the slope and maximum discharge of pressure discharge relations of regularly discharging BRs (Figure 4). In half the neurons tested, these effects reversed poorly with persistent depression lasting through extended periods of perfusion in CAP-free solution (>30 min).

**Figure 5:**
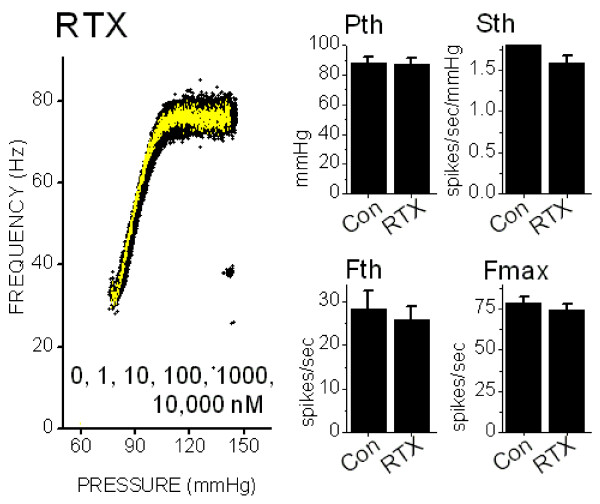
Resinferotoxin (RTX) did not alter the pressure-discharge relations of regularly discharging, CAP-resistant BRs. On the left, the pressure-discharge relations for a representative, regularly discharging BR (COV = 1.9%, n = 677 points) that was exposed to RTX increasing concentrations (1, 10, 100, 1000, 10,000 nM) for 15 min each. The five pressure-discharge relations during RTX closely overlaid the original control relation (left panel). Instantaneous discharge values during 10,000 nM are colored yellow and all other datasets are black. The relationships completely overlapped. Average values for the discharge parameters (histograms on right) for seven regularly discharging BRs before and after 10,000 nM RTX are plotted. Pressure threshold (Pth), threshold frequency (Fth), slope sensitivity (Sth) and the maximum discharge frequency (Fmax) are displayed. Thus, unlike CAP, very high concentrations of RTX did not produce nonspecific effects on BR discharge.

The ultrapotent RTX may be effective at >100x lower concentrations than CAP[21]. Here, we tested RTX actions on pressure-evoked BR discharge. RTX at concentrations from 1 nM to 10 μM had no significant effects on pressure-discharge relations of regularly discharging, A-type single unit BRs. On average (n = 7, Figure 5), 1 μM RTX did not alter Pth, Sth, Fth, or Fmax (p > 0.18, n = 7). In one BR, RTX concentration was increased to 10 μM RTX (Figure 5, left), a level that is more than 1000 times higher than the expected ED50 without "nonspecific" effects on pressure-discharge relations. This finding contrasts with the frank depression of A-type BR pressure-discharge relations found with high CAP concentrations (Figure 4).

## Discussion

The rat ADN is a unique peripheral nerve trunk since it contains only aortic BR afferent axons [22-24]. This thin ADN nerve bundle (~ 100 μm diameter) consists of up to 80% C-fibers of the total axon population [11,25,26] without the efferent axons found in the peripheral vagus or somatic nerve trunks. This anatomical feature means that, experimentally, ENGs evoked by electrical stimulation are responses to a relatively uniform modality of primary visceral afferents – in this case BRs – and should not be influenced by contributions from antidromically activated efferent (non-sensory) axons of similar conduction velocity that can be a complication in recordings from a mixed (afferent-efferent) nerve trunk. The close correspondence in our CAP results between the population ENG responses and our single fiber BR studies is consistent with this homogeneity. Our studies suggest two important findings about these cranial visceral afferents and their expression of TRPV1. First, aortic BRs possess CAP-resistant and CAP-sensitive peripheral nerve endings and the site of action is presumably close to or within the mechanoreceptive fields at the aortic arch wall. Second, very high concentrations of CAP but not RTX (>10 μM) have additional inhibitory actions on these neurons that are consistent with non-specific effects.

A-type BR fibers were strongly resistant to CAP actions, both along their peripheral axons as well as in the pressure transduction process at their peripheral endings at the aortic arch. The CAP resistant component of the whole nerve measurements during ADN conduction was within the Aδ class of BR axons – simply designated A-type. Such A-type BRs are likely typical of a broad association for cranial visceral afferent neurons with CAP sensitivity. It was recently reported that nodose neurons with A-fiber conduction velocities did not respond to somatic exposure to CAP whereas C-fiber neurons were CAP-sensitive [27]. In addition, afferent synaptic transmission was block to some neurons within the central nervous system to neurons within the nucleus of the solitary tract (NTS)[27,28]. Together, our present peripheral nerve studies together with nodose ganglion and NTS studies indicate that TRPV1 is expressed in C-type, primary visceral afferent neurons at their cell body membrane as well as at both their peripheral sensory endings and central synaptic terminals.

In the present studies, we successfully recorded from only one single fiber BR with the irregular discharge pattern consistent with an unmyelinated BR [29,30]. Axon length was insufficient to measure the conduction velocity, but nonetheless, pressure-discharge responses in this highly irregular BR were blocked by 1 μM CAP – a finding consistent with ADN whole nerve recordings and previous studies of rat ADN C-fiber BRs [29-31]. Like other BR modulating agents, lumenal perfusion with CAP rapidly influenced BR discharge properties despite the location of the BR endings within the adventitia [14,16]. Despite these limitations of the single fiber BR recordings, the ADN ENG results bolster the association of C-type BRs with CAP sensitivity and A-type BRs with CAP resistance consistent with other studies [27].

CAP has long been reported to have "non-specific" actions on neurons and other cell types that are not likely to involve activation of TRPV1 [20]. Chronic toxicity is likely related to prolonged activation of TRPV1 that may lead to calcium overload [32] but it should be noted that these toxic actions require that the affected neuron express TRPV1. In our case of "non-specific" actions, we presume that A-type BRs lack TRPV1 expression but nonetheless have discharge depressed by high concentrations of CAP – that is, a TRPV1-independent action of CAP. Concentrations of substances delivered to the aortic lumen appear to faithfully reach the BR endings at the expected concentrations [14]. The concentration-response profile for our pledget application of CAP applied to the axon trunk suggests that this method of delivery may be highly inefficient and concentrations at the site of action may be much lower than applied concentrations [15]. Thus, 10 μM CAP blocked C-fiber conduction in the ADN within 10 min but appeared to be specific in that A-fibers were not blocked [33]. However, 1 μM CAP show similar C-selective axonal block but required 30 min in some ADNs.

For single fiber BR discharge, the qualitative pattern of the blockade by CAP was similar to other highly specific agents that modulate BR transduction [14,16,34,35]. In the CAP-sensitive BR, the upper limits of discharge frequency changed substantially during CAP. The relations did not shift along the pressure axis as is found for sodium channel blockade with TTX in BRs [14,16]. Less selective agents than TTX that are known to act at both sodium and potassium channels such as local anesthetics [14,16,36]. Likewise, changes in extracellular ion gradients[16,37] change threshold parameters as well as slope sensitivities of BRs. Thus, in the case of the CAP-sensitive BR responses, the transduction of pressure appeared to remain intact since the threshold and slope sensitivity to pressure were constant until final block developed. However in A-type BRs, the high concentrations of CAP produced multiple, global changes in the pressure-discharge relations, changes that are consistent with multiple targets of action. Interestingly, RTX, a high affinity ligand for VR1, did alter regularly discharging BRs even at higher relative concentrations [20]. The broader impact on BR discharge characteristics at very high concentrations conceivably could be a consequence CAP perturbation of lipid-protein interactions such as ion channels[20]. Thus, non-specific actions of CAP may depress a range of ion channels indirectly in A-type BRs lacking TRPV1 expression.

Our whole-nerve ADN recordings provide conduction velocity information on the population of ADN BR primary afferents. In this respect, no BR in our single fiber BR studies should have a conduction velocity beyond 12 m/sec – a value that corresponds to the lightly myelinated Aδ class of fibers[4,38]. The broad ENG profile of the C-fiber volley suggests that ADN non-myelinated fibers range in conduction velocity from 1 m/sec to as low as 0.2 m/sec. Within these limits, ADN BRs conduct within similar ranges of both the Aδ and C-fiber nociceptive fibers of the DRG [39]. Presumably, BR neurons would be among the neurons expressing TRPV1 mRNA at the NG [9,40]. VR1 protein is present in the medial portions of the NTS in which BR sensory neurons have synaptic terminations [41,42]. CAP evoked somatic inward currents only in NG neurons recorded in slices with the with conduction velocities in the C-fiber range and A-fiber NG neurons were CAP-resistant[27]. Together, the evidence suggests that TRPV1 is present only in cranial visceral primary afferents with C-fiber axon type and that CAP-resistant BRs have A-type conducting axons and lack TRPV1 expression.

Overall, A-type aortic BR neurons appear devoid of functional TRPV1. C-type aortic BRs express TRPV1 in their peripheral axons and in their aortic arch terminations and appear to be part of a broader group of cranial visceral afferents that are known to be CAP-sensitive and centrally provide for respiratory and gastrointestinal as well as cardiovascular regulation [43,44]. Interestingly, activation of aortic BRs and the arterial baroreflex evoke anti-nociceptive responses in animals and in humans[45,46]. Ultimately, the role of TRPV1 is unclear in cranial visceral afferents but conceivably could contribute to surveillance of tissue damage [47].

## Conclusion

We conclude that functional TRPV1 channels are not present in A-δ myelinated BRs at their aortic arch endings or peripheral axons. CAP, unlike the ultrapotent TRPV1 agonist RTX, had additional non-specific actions to inhibit discharge of pressure activated A-type aortic BRS at very high concentrations. Thus, CAP appears to have toxic actions at high concentrations that are independent of TRV1 expression and must be used with caution in experiments in which concentration is not well controlled.

## Methods

All animal procedures were conducted with the approval of the Institutional Animal Care and Use Committee in accordance with the U.S. Public Health Service Policy on Humane Care and Use of Laboratory Animals (PHS Policy) and the National Institutes of Health Guide for the Care and Use of Laboratory Animals (NIH Guide). Experiments used adult male Sprague-Dawley rats (B & K, Inc., Kent, Washington, 250–450 g).

### Isolation of the left aortic depressor nerve

Rats were anesthetized with pentobarbital sodium (ip 35–50 mg/kg). The methods of the fine dissection to isolate the left ADN as well as removing the ADN with the aortic arch intact have been described in detail previously[25]. Briefly, following a ventral midline incision in the neck, the trachea was intubated and connected to a ventilator. The chest was opened at the midline along the sternum and the chest wall retracted to expose the heart and aortic arch. From the NG to the aortic arch, the ADN was microdissected under 20–60x magnification of a stereomicroscope (Wild 6A, Heerbrugg, CH). In whole nerve trunk experiments, the ADN was sectioned close to the aortic arch and near the NG recorded in situ [15] with distal nerve stimulation and recording of the evoked whole-nerve, compound action potential through hook electrodes under oil. For recording single BR discharge from the in vitro ADN aortic arch preparation, the aortic arch was cannulated and removed with the ADN for mounting in a perfusion-recording chamber.

### Compound action potentials in ADN

Isolation of sufficient lengths of ADN in order to measure conduction velocity of the fastest conducting fibers is difficult [15]. Here, bipolar electrode pairs (Teflon-coated Pt-Ir wires) were placed on both the rostral and caudal cut ends of the ADN trunk. The path distance for conduction between them was measured with an ocular micrometer of the stereomicroscope at 36 × magnification. The nerve and electrodes were then covered with a mixture of petroleum jelly and warm mineral oil for isolation. Electrical stimuli were delivered via one electrode pair and the other pair recorded evoked compound action potentials. The stimulating electrodes were connected to a computer-controlled programmable stimulator (AMPI Master-8) through a stimulus isolation unit. CAP or vehicle was delivered to the axon bundle at an exposed region between the two sets of electrodes using small pieces of solution soaked cotton[15]. Threshold stimulus intensities were established in each preparation. Shocks were 0.1 msec in pulse duration. Supramaximal stimulus intensities consistently activated similar electroneurogram (ENG) waves in the ADN when the nerve was stimulated once each 30 sec. Compound action potentials were captured for analysis using either a digital oscilloscope (Hewlett-Packard 54645A, Agilent Technologies, Palo Alto, CA) or computer (Datawave Technologies, Longmont, CO). Components of the compound action potentials were compared before and after VR1 agonist treatment by full wave rectification followed by integration (Figure 1). These integrals measured the selected wave component over a 10 msec interval centered on the A-wave and over a 50 msec interval centered on the complex C-wave. The time of appearance within the ENG corresponded to the range of conduction velocities.

### *In-vitro *BR pressure-discharge recording

The aortic arch with attached ADN was placed within a temperature-regulated (36–37°C) chamber to provide a stable controlled preparation in which highly reproducible pressure stimuli were delivered through the stainless steel cannulae of the perfusion system. The arch was continuously perfused (3 ml/min) with a physiological buffered salt solution (PBS). PBS contained the following in mM: 120 NaCl, 5 KCl, 1.2 K_2_HPO_4_, 1.2 MgSO_4_, 1.2 CaCl_2_, 25 NaHCO_3_, and 10 glucose, and was continuously bubbled with a 95% O_2_–5% CO_2 _gas mixture. All drugs were added to the perfusate. Aortic arch pressure was measured via a side port manometer, displayed on a chart recorder and recorded on FM magnetic tape. Aortic arch pressure was maintained at a constant non-pulsatile 80 mmHg between tests.

### Single fiber BR pressure tests

For single fiber studies, whole ADN nerves were split to fine filaments by successive micro-dissection using 30G disposable needles (Bectin Dickinson, Franklin, NJ). Those splits containing one or a few active fibers were placed on a pair of fine Pt-Ir electrodes that were connected to a high-gain differential preamplifier (PAR 113, Princeton E, G & G). Unit discharge responses were recorded during slow ramp pressure tests (<2 mmHg/sec). For each test (control and drug trials), a series of three pressure ramps at five-minute intervals was applied to the arch and the full pressure response curve was recorded. For offline analysis, pressure was sampled at 100 Hz and the instantaneous spike frequency was calculated from pulses from a time-amplitude window discriminator (DIS-1, Bak Electronics, Mount Airy, MD) by a custom software program on a PC-based computer system. Final data analyses and comparisons focused on the third (15 min) trials for each condition.

### BR discharge properties – distinguishing types

Short lengths that were split for single fiber recordings from ADN did not permit measurements of conduction velocity in these BR unit studies. However, several characteristics of the distribution of interspike intervals and the pressure-discharge relations distinguish two classes of discharge pattern, regular and irregular, that are associated with A- and C-type BRs, respectively [13,29,31,48-50]. Individual A-type BRs fire at highly regular interspike intervals whereas C-fiber BRs in contrast fire very irregularly [51]. Discharge frequency was calculated as the inverse of the interspike interval, that is the instantaneous discharge rate[14]. The variation in this instantaneous discharge frequency served as a key discriminating indicator between BR fiber types[13]. Variability in interspike interval was expressed as the coefficient of variation (COV) of the discharge intervals for each BR unit and calculated as the ratio of the standard deviation of the discharge rate divided by the mean discharge rate. The COV was calculated from segments of the control pressure-discharge relations over a fixed range of 10 mm Hg (from 140 to 150 mmHg) during the pressure ramp. For most units, these pressures were within the plateau range of pressures at near maximum discharge frequency. This COV index was used to distinguish regularly from irregularly discharging BRs.

### Analysis – pressure-discharge curves

To characterize BR pressure-discharge relations, instantaneous frequency was plotted versus mean arterial pressure (OriginLabs, Origin 7.5, Northampton, MA) and characteristic discharge parameters were derived from the plots for each unit. A-type BRs exhibited a sharp jump from zero to a set frequency of regular discharge once they exceeded an adequate activation pressure, the threshold pressure (Pth)[13,18]. When averaged over the first ten points, that frequency of discharge at threshold (Fth) was highly consistent across repeated trials over time. The pressure-frequency relation above Pth was highly linear. The slope of the relation defined the suprathreshold pressure sensitivity (Sth or gain) and could be measured by fitting with a linear regression (r^2 ^>0.9) over an interval of 20–40 mmHg before reaching a plateau of maximum discharge frequency (Fmax, calculated as an average frequency across 20 mmHg and 200–250 spikes). C-type BRs, in contrast, have generally higher Pth and lower Fth, Fmax, and Sth as well as higher COV. Characteristic discharge parameters (Pth, Fth, Sth, and Fmax) were derived from the plots for each unit. Parameters from the 15 min tests for each condition were compared to controls by analysis of variance. P-values less than 0.05 were considered to be significant.

### VR1 agonists

CAP was initially dissolved in DMSO and then mixed with PBS to a concentrated stock solution containing 300 μM CAP. This CAP stock solution was diluted with PBS to the final concentrations perfused through the aortic arch lumen. Controls included trials with application of vehicle alone at the highest concentration employed (0.1% DMSO). Dose response relations were difficult to obtain with CAP due to the slow time course of recovery and the likelihood of desensitization [33]. Since we were most interested in establishing whether BRs were sensitive to TRPV1 agonists, we tested BRs at a CAP concentration of 1 μM, a concentration chosen to be sufficient to saturate TRPV1 and yet still remain within the "selective" concentration range described by others [52]. Furthermore, to test this assumption, we extended the CAP concentration up to 100 μM to test for "non-specific" CAP effects in some experiments. RTX was initially dissolved in a concentrated stock solution as a mixture of methyl alcohol (<1%) and PBS containing 50 μM RTX. This RTX stock solution was diluted with PBS to final test concentrations. Controls included trials with vehicle alone. We applied RTX over a concentration range from 1 nM to 10 μM. RTX concentration of 1 nM was presumed to be a saturating concentration for TRPV1 [53] and >1 nM were applied to test for possible "non-specific" RTX effects.

## Competing interests

The author(s) declare that they have no competing interests.

## Authors' contributions

PR and WF carried out the single fiber studies plus their analysis and participated in the drafting of the manuscript. MA conceived and designed the study and carried out the whole nerve recordings. MA analyzed the digital recordings as well as drafted the manuscript. All authors read and approved the final manuscript.
